# Assessment of the risk associated with *Bacillus cereus* isolates and potential combat with methanolic *Artemisia vulgaris* extract

**DOI:** 10.3389/fmicb.2025.1640200

**Published:** 2025-10-28

**Authors:** Mohamed A. Fareid, Gamal M. El-Sherbiny, Nancy M. Elafandy, Nagat E. Eltoum, Mohamed S. Othman, Mohamed H. Sharaf, Mohammed Abu-Elghait, Dina M. Elkhashab, Fatma A. Hamada

**Affiliations:** ^1^Clinical Laboratory Science Department, Applied Medical Science College, University of Ha'il, Hail, Saudi Arabia; ^2^Botany and Microbiology Department, Faculty of Science, Al-Azhar University, Cairo, Egypt; ^3^Clinical Nutrition Department, Applied Medical Science College, University of Ha'il, Hail, Saudi Arabia; ^4^Biochemistry Department, College of Medicine, University of Ha'il, Hail, Saudi Arabia; ^5^Clinical Pathology Department, National Cancer Institute, Cairo university, Cairo, Egypt; ^6^Basic Sciences Department, First Year of Health and Medical Colleges, University of Ha'il, Hail, Saudi Arabia

**Keywords:** *B. cereus*, foodstuffs, virulence genes, antibiotic resistance, biofilm, *Artemisia vulgaris* extract, antibacterial activity, antioxidant

## Abstract

**Introduction:**

*Bacillus cereus (B. cereus)* is widely distributed in natural environments, particularly in soil and plant matter, and is frequently linked to foodborne illness outbreaks, accounting for approximately 1.4%–12% of food poisoning cases worldwide. This study aimed to assess the presence of toxigenic and emetic genes among *B. cereus* isolates, evaluate their antibiotic susceptibility, and investigate the antimicrobial potential of *Artemisia vulgaris (A. vulgaris)* extract.

**Methods:**

Polymerase chain reaction (PCR) was employed to detect toxigenic and emetic genes in *B. cereus* isolates. Antibiotic susceptibility was tested against a panel of agents. The antimicrobial, antibiofilm, antioxidant, cytotoxic, and gene-suppressing activities of methanolic *A. vulgaris* extract were evaluated using standard microbiological, biochemical, and molecular assays. GC-MS and HPLC analyses were performed to identify major bioactive compounds.

**Results:**

PCR revealed that the isolates harbored hemolysin BL (HBL) genes *hblA* (8.62%) and *hblB* (20.68%), non-hemolytic enterotoxin (NHE) genes *nheA* (20.68%) and *nheB* (22.41%), as well as *bceT* (29.30%) and *ces* (15.51%) genes associated with emetic toxin production. Antibiotic testing showed high sensitivity to ciprofloxacin (91.37%) and rifampicin (96.54%), but strong resistance to ampicillin (86.20%) and novobiocin (65.51%). A. vulgaris extract demonstrated potent antibacterial activity (inhibition zones: 19.20 ± 0.25 mm to 27.10 ± 0.13 mm; MICs: 62.5–250 μg/mL), significantly inhibited biofilm formation, and downregulated toxigenic genes by −2.5 to −5.2 fold (^***^P < 0.0001). The extract also displayed strong antioxidant activity (IC_50_: 12.7 μg/mL, DPPH; 14.2 μg/mL, ABTS) and low cytotoxicity (IC_50_: 524.7 ± 1.23 μg/mL, Vero cells; 236.5 ± 1.74 μg/mL, HFB4 cells). GC-MS identified dopamine N,N-dimethyl-dimethyl ether (40.31%) and n-hexadecanoic acid (16.57%) as major compounds, while HPLC revealed high levels of chlorogenic acid, luteolin, and quercitrin.

**Discussion/conclusion:**

These findings highlight the public health risks posed by toxigenic *B. cereus* in food contamination. The methanolic extract of *A. vulgaris* exhibits strong antibacterial, antibiofilm, antioxidant, and gene-suppressing activities, supporting its potential as a natural therapeutic strategy against *B. cereus* and its virulence factors.

## 1 Introduction

*Bacillus cereus* is a motile, rod-shaped bacterium that is Gram-positive and can thrive in both aerobic and facultatively anaerobic conditions (Bottone, 2010). *B. cereus* is categorized within the *B. cereus* sensu lato group, which includes other bacteria such as *B. anthracis, B. thuringiensis, B. mycoides, B. pseudomycoides, B. weihenstephanensis, B. cytotoxicus*, and *B. toyonensis*, as well as several newly discovered species identified through modern genetic taxonomic research, including *B. gaemokensis, B. manliponensis*, and *B. bingmayongensis*. A significant genetic resemblance is observed among these bacterial species (Enosi Tuipulotu et al., 2021). Due to the significant differences in the growth and survival characteristics of *B. cereus* strains, they are classified into two groups: mesophilic and psychrotrophic. The psychrotrophic strains thrive at temperatures below 10 °C, but they do not perform well at 37 °C. These strains may sometimes be found in fresh foods, although they are more commonly observed in cold dishes. In contrast, mesophilic strains can endure temperatures below 10 °C and prosper at 37 °C (Webb et al., 2019).

Through the production of endospores and the formation of biofilms, *B. cereus* is capable of surviving and flourishing in challenging environments. The elongated spores of *B. cereus* are encased by a peptidoglycan cortex, an inner membrane, an outer coat, and an inner coat. These bacterial spores, which exhibit resistance to heat, freezing, desiccation, gamma radiation, and UV radiation, remain metabolically inactive. Their remarkable resilience to environmental factors that typically eliminate vegetative bacteria enables them to withstand the emergence of more favorable conditions (Bressuire-Isoard et al., 2018; Kailas et al., 2011; Duport et al., 2016). They also facilitate adherence to human epithelial cells. Both living tissues and non-living surfaces can sustain *B. cereus* biofilms, which may also manifest as floating pellicles. These biofilms are linked to nosocomial bacteremia and have been observed on central venous catheters. Notably, in contrast to planktonic cells, *B. cereus* within biofilms produces a greater quantity of secondary metabolites, including catalase and superoxide dismutases, thereby enhancing the bacteria's resistance to host immune responses (Vidic et al., 2020; Ikram et al., 2019; Caro-Astorga et al., 2020).

Food poisoning incidents, which generally appear as emetic and/or diarrheal syndromes, can result from the contamination of food products by bacteria associated with the *B. cereus* group (Gdoura-Ben Amor et al., 2019; Bennett et al., 2013; Stenfors Arnesen et al., 2008). Consuming cereulide, a preformed toxin present in tainted food, is linked to the emetic form of food poisoning. The *ces* gene encodes this toxin, a cyclic dodecadepsipeptide that is stable in the face of heat and pH changes and resistant to proteolytic enzymes, which keeps it active throughout the gastrointestinal tract. One or more heat-labile enterotoxins generated in the small intestine are responsible for the diarrheal form of food poisoning (Ceuppens et al., 2012; Hwang and Park, 2010). NHE, encoded by *nheA, nheB*, and *nheC*; HBL encoded by *hblA, hblB, hblC*, and *hblD;* and cytotoxin K, encoded by *cytK*, are the enterotoxins associated with *B. cereus* group bacteria that are mostly in charge of diarrheal illnesses (Gdoura-Ben Amor et al., 2019; Zhang et al., 2015). Guinebretiere et al. (2006) and Castiaux et al. (2015), have reported two different forms of *CytK*, which they have named *cytK-1* and *cytK-2*. Despite having an 89% protein sequence similarity to *CytK-2, CytK-1* is much more hazardous. Among the diarrhoeal enterotoxins include enterotoxin T, which is encoded by the *bceT* gene, in addition to HBL, NHE, and *CytK*. Subsequent research has raised questions about the activity and classification of *bceT* enterotoxin as an enterotoxin, and its function in food poisoning has not been proven (Gdoura-Ben Amor et al., 2019; Choma and Granum, 2002; Hansen et al., 2003). The *bceT* gene's product has been hypothesized to be a cloning artifact because it has no biological function and does not cause epidemics. The degree of virulent gene expression determines the true risk of food poisoning linked to the *B. cereus* group. Bacterial cell concentrations of 5 to 8 log_10_ CFU/g and 5 to 7 log_10_ CFU/g, respectively, can cause diarrheal and emesis symptoms. As a result, food manufacturers are generally advised to classify foods that contain 10^5^ CFU/g of *B. cereus* as unfit for human consumption (Gdoura-Ben Amor et al., 2019; Ceuppens et al., 2011). *B. cereus*-related food poisoning outbreaks have been reported globally. In the European Union, *B. cereus* toxins were identified as the leading cause of food poisoning outbreaks in 2020, leading to 71 outbreaks, 835 cases, 10 hospitalizations, and one fatality. In Southern Brazil, *B. cereus* was the predominant pathogen associated with foodborne illnesses from 2003 to 2013. In China, *B. cereus* emerged as one of the most frequently confirmed pathogens responsible for foodborne outbreaks in recent years. On May 12, 2021, an outbreak of gastrointestinal illness was documented in two middle schools located in a rural region of Chongqing, China. Over one hundred students from both schools exhibited symptoms of vomiting and nausea, which prompted the Chongqing Center for Disease Control and Prevention (CDC) and the local CDC to initiate an investigation and implement necessary control measures (Li et al., 2023).

*A. vulgaris* belongs to the *Compositae* family and historically has been extensively employed in various cultures owing to its anthelmintic, antiseptic, antimicrobial, antidiabetic, antidepressant, and vermicidal attributes (Pandey et al., 2017). A diverse range of bioactive secondary metabolites, including essential oils, flavonoids, triterpenes, and coumarin, constitute the primary components of the *A. vulgaris*. The essential oils derived from *A. vulgaris* exhibited an inhibition zone measuring 15 mm against *Acinetobacter baumannii* ATCC 19606, 12 mm against *Klebsiella pneumonia* ATCC 700603, and 11 mm against *Staphylococcus aureus* ATCC 43300 (Sharma, 2023). *A. vulgaris* essential oil showed antibacterial activity against *S. aureus* (ATCC 25923) and *B. cereus* (ATCC 13061). For *B. cereus* and *S. aureus*, the essential oil's MIC and minimum bactericidal concentration (MBC) were found to be 0.3% and 0.63%, respectively (Singh et al., 2023).

Although several research studies have explored the antimicrobial characteristics of *A. vulgaris*, this study is unique as it represents the first instance of its application against *B. cereus* and the risks it poses. This pioneering approach may open new avenues for developing natural antimicrobial agents, potentially reducing reliance on synthetic alternatives. As the prevalence of antibiotic resistance grows, findings from this research could be crucial in shaping future therapeutic strategies. Therefore, the purpose of this study was to assess the toxigenic characteristics of *B. cereus* isolates and potential fighting with *A. vulgar*is extract as a promising alternative approach.

## 2 Materials and methods

### 2.1 Chemicals and reagents

The culture media, including Muller Hinton broth (MHB) and Muller Hinton agar (MHA), were purchased from Himedia (India), while chemicals and reagents, including methanol, chloroform, ethyl acetate, and antioxidant materials, including diphenyl-1-picrylhydrazyl (DPPH), 2,2′-azino-bis [3-ethylbenzothiazoline-6-sulfonic acid (ABTS)], potassium persulfate, and ascorbic acid, were obtained from Sigma-Aldrich in St. Louis, MO, USA.

### 2.2 Plant material

*A. vulgaris* leaves were obtained from a local market in Ha'il, Saudi Arabia, in March 2025. The leaves were thoroughly washed under running tap water to remove any impurities, followed by rinsing with distilled water. They were then placed on clean paper towels and left to dry at 25 ± 2 °C for three days. After drying, the leaves were ground into a fine powder using a high-speed blender for 5 min. The resulting powder was passed through a 40-mesh sieve to ensure uniform particle size. A total of 400 g of powdered *A. vulgaris* leaves were divided into four equal portions of 100 g each. Each individual portion of 100 g was extracted separately using a single solvent (the first portion was extracted with 500 ml of methanol, the second portion with 500 ml of water, the third portion with 500 ml of chloroform, and the fourth portion with 500 ml of ethyl acetate), utilizing a Soxhlet apparatus (Model No. 3840, Borosil Glass Works Ltd., Mumbai, India) at a temperature of 50 °C for a duration of 8 h. The extracts were then filtered through Whatman No. 1 filter paper, and the solvents were evaporated under reduced pressure using a rotary vacuum. The remaining extracts were dried and stored at −4 °C in a refrigerator for subsequent studies.

### 2.3 *B. cereus* isolates

Fifty-eight *B. cereus* isolates were isolated from various samples, including cereals, spices, canned goods, pastry items, and fresh-cut vegetables, purified, and identified using the Vitek2 system (GP-card) Version 05.04, produced by BioMerieux SA, France. These isolates were obtained from the bacteriology laboratory at the Faculty of Science, Al-Azhar University, Cairo, Egypt.

#### 2.3.1 DNA extraction

For DNA extraction, the *B*. *cereus* isolates were cultivated on MHB medium for 20 h at 30 °C without stirring. A 15 ml Falcon tube containing 300 μl of a 25% (m/v) sterile Chelex bead solution (Saint-Herblain, France) made in sterile Milli-Q water (Sigma Aldrich) was then filled with 5 ml of each culture bacterial isolate. After vortexing, the mixture was centrifuged for 7 min at 4 °C at 7,000 rpm. After that, the cell pellet was resuspended in 200 μl of sterile Milli-Q water and heated for 10 min at 100 °C to lyse it. One hundred and fifty microliter of the supernatant was taken and centrifuged again under the same condition following a second centrifugation at 7,000 rpm for 7 min at 4 °C (Walsh et al., 1991). A NanoDrop ND-1000 spectrophotometer (Nanodrop Technologies, Wilmington, USA) was used to measure DNA concentration. The sample was then diluted until it reached a final concentration of about 100 ng/μl.

#### 2.3.2 Detection of toxigenic genes

The emetic (*ces*) and enterotoxigenic genes (*hblA, hblB, nheA, nheB*, and *bceT*) were investigated in the *B. cereus* isolates. The primers were employed with their annealing temperatures, and amplified fragments for each gene are presented in Table 1. Each reaction mixture contained 5 μl of 100 ng DNA template, 2 μl of each primer (Sigma Aldrich) at a concentration of 10 μM, 0.3 μl of Taq polymerase (5,000 U/ml) (Biolabs, Evry, France), 0.5 μl of 10 mM deoxyribonucleotide triphosphate (Eurogentec, Seraing, Belgium), 1.12 μl of 50 mM MgCl_2_ (Biolabs), 2.5 μl of 10X AmpliTaq buffer (Biolabs), and 14.5 μl of sterile Milli-Q water. Thermocycleur PCR (Biosystems, USA) was used to perform amplification reactions. The amplification parameters for the *ces* cluster were as follows: 5 min at 95 °C, 30 cycles of 15 s at 95 °C, 30 s at 58 °C, and 30 s at 72 °C, and an 8-min final extension at 72 °C. Two minutes at 94 °C, ten cycles of 10 s at 94 °C, 30 s at 58 °C, and two min at 68 °C were the amplification conditions for *hblB*. Twenty cycles of 10 s at 94 °C, 30 s at 58 °C, and 2 min (plus an extra 20 s per cycle) at 68 °C were performed after these ten cycles, with a final extension at 68 °C lasting 7 min (Ehling-Schulz et al., 2004, 2005; Guinebretière et al., 2002). The amplification conditions for the other toxin genes (*hblA, nheA, nheB*, and *bceT*) were as follows: 4 min at 95 °C, 30 cycles of 30 s at 95 °C, 30 s at the corresponding annealing temperatures as indicated in Table 1, and 1 min at 72 °C, culminating in a final extension of 7 min at 72 °C. A negative control comprising the entire PCR mixture devoid of any DNA template was included in every run (Techer et al., 2014).

**Table 1:** Primers are employed to detect the toxigenic and emetic genes in *B. cereus*.

**Targeted gene**	**Primer**	**Sequence (5^′^-3^′^)**	**Product size (bp)**	**Annealing temp (°C)**	**References**
*hblA*	HA F	AAGCAATGGAATACAATGGG	1,154	56	Ehling-Schulz et al., 2004
	HA R	AGAATCTAAATCATGCCACTGC			
*hblB*	HB F	AAGCAATGGAATACAATGGG	2,684	58	Ehling-Schulz et al., 2004
	HB R	AATATGTCCCAGTACACCCG			
*nheA*	NA F	GTTAGGATCACAATCACCGC	755	56	Ehling-Schulz et al., 2004
	NA R	ACGAATGTAATTTGAGTCGC			
*nheB*	NB F	TTTAGTAGTGGATCTGTACGC	743	54	Ehling-Schulz et al., 2004
	NB R	TTAATGTTCGTTAATCCTGC			
*bceT*	bceT F	GCTACGCAAAAACCGAGTGGTG	679	57	Gdoura-Ben Amor et al., 2019
	bceT R	AATGCTCCGGACTATGCTGACG			
*ces*	EM1F	GACAAGAGAAATTTCTACGAGCAAGTAAT	635	58	Ehling-Schulz et al., 2005
	EM1R	GCAGCCTTCCAATTACTCCTTCTGCCACAGT			
	Ces F	GGTGACACATTATCATATAAGGTG	1,271	5	Techer et al., 2014
	Ces R	GTAAGCGAACCTGTCTGTAACAACA			

### 2.4 Antibiotic susceptibility assay

The disc diffusion technique was used to assess the susceptibility of *B. cereus* isolates to fifteen different antibiotics, including ciprofloxacin (5 μg), amikacin (30 μg), chloramphenicol (30 μg), novobiocin (30 μg), fusidic acid (10 μg/ml), streptomycin (10 μg), kanamycin (30 μg), ampicillin (10 μg), vancomycin (30 μg), oxacillin (1 μg), gentamicin (10 μg), nitrofuran (50 μg), erythromycin (15 μg), tetracycline (30 μg), and rifampicin (5 μg) (Oxoid, England). A 100 μl of *B. cereus* isolates freshly prepared at 0.5 McFarland were spread on the MHA surface and loaded with antibiotic discs. For 20–24 h, the plates were incubated at incubated at 30 °C. The inhibition zone was measured and interpreted according to guidelines established by the Clinical and Laboratory Standards Institute (CLSI) (CLSI, 2023).

### 2.5 Antibacterial activity of *A. vulgaris* extracts against *B. cereus* isolates

To evaluate the antibacterial activity of *A. vulgaris* leaf extracts, the six highly resistant *B. cereus* isolates were cultivated in MHB and incubated for 20 h at 37 °C. A 100 μl of bacterial suspension adjusted to a McFarland turbidity of 0.5 was spread on MHA surface plates. After inoculating the MHA with the freshly prepared bacterial isolates, wells of 6 mm in diameter were created using a sterile cork borer. Each well was filled with 100 μl of *A. vulgaris* extract at a concentration of 150 μg/ml, along with a gentamicin disc (10 μg/mL) as a positive control, and the plates were incubated for 2 h at 4 °C. Following a 24-h incubation period at 37 °C, the zones of inhibition on the plates were measured with (mm). The experiment was conducted in triplicate (Foda et al., 2022; Sharaf et al., 2021).

### 2.6 Minimum inhibitory concentration of methanolic *A. vulgaris* extract against *B. cereus* isolates

The MIC values of ciprofloxacin (HiMedia Laboratories Pvt. Ltd. India.) and highly active extract (methanolic *A. vulgaris* extract) were determined using microdilution assay in a 96 well plate. Muller Hinton broth medium was inoculated with a cell suspension of *B. cereus* isolates that had been calibrated to a concentration of (10^6^ CFU/ml) and 200 μl of the inoculated medium was distributed in each well. Tested compounds (ciprofloxacin and methanolic *A. vulgaris* extract) were tested in a 2-fold serial dilution. Ciprofloxacin was tested at concentrations ranged from 0.1 to 10.0 μg/ml and methanolic *A. vulgaris* extract started with 0.0 to 1,000.0 μg/ml. The assay was performed according to the criteria of M7-A7. Wells containing negative control (medium + ciprofloxacin or methanolic *A. vulgaris* extract at the tested concentrations) were performed to determine the differences in optical density. The plates were incubated for 20 h at 30 °C and the absorbance was measured at 630 nm. MIC was defined as the lowest concentration of the ciprofloxacin or methanolic *A. vulgaris* extract which is able to inhibit the visible growth of *B. cereus* isolates (CLSI, 2023; El-Sherbiny et al., 2024).

### 2.7 Antibiofilm activity of methanolic *A. vulgaris* leaves extract

The efficacy of the methanolic extract of *A. vulgaris* leaves in preventing the formation of biofilms by *B. cereus* isolates was quantitatively assessed using the microplate assay. The *B. cereus* isolates were cultivated on MHB for 20 h. A 100 μl of a bacterial suspension adjusted to 0.5 McFarland turbidity was added to each well. The wells were filled with 100 microliters of methanolic extract of *A. vulgaris* leaves at three different concentrations (1/8, ¼, and ½ MIC). The experiment included wells with control of growth media without inoculum, methanolic extract, and growth media; wells containing growth media and bacterial suspension (untreated); and incubation at 37 °C for 24 h. The optical density (OD) was measured at 630 nm. The following formula was used to determine the percentage of biofilm inhibition: % inhibition = [(OD negative control – OD medium control) – (OD test – OD *A. vulgaris* control)]/(OD medium control – OD negative control) × 100 (Fareid et al., 2025). Three duplicates of the experiment were conducted, and the mean ± standard deviation was used to report the results.

### 2.8 Effects of methanolic *A. vulgaris* extract on expression of toxigenic and emetic genes in *B. cereus* isolates

A suspension of *B. cereus* isolates grown on MHB medium was prepared at a 0.5 McFarland concentration and then subjected to treatment with half the MIC of methanolic *A. vulgaris* extract, while untreated bacterial strains were used as a control. The cultures were incubated at 37 °C for 24 h. The expression levels of the *hblA, hblB, nheA, nheB, bceT*, and *ces* genes were analyzed using qRT-PCR, with the primer sequences provided in Table 1. cDNA synthesis was performed using the AMV reverse transcriptase enzyme (Roche, Basel, Switzerland) at a concentration of 25 units/L, along with transcriptase. To avoid the formation of secondary structures, the RNA extracted in the previous step was heated to 65 °C for 3 min. Reverse transcription (RT) was conducted at 42 °C for 60 min, utilizing 2 μL of random primer and 2 × AMV reverse transcriptase enzymes, followed by incubation and inactivation of the AMV enzyme at 99 °C for 5 min. Gene expression quantification was executed using the 2^−ΔΔCT^ method (Guinebretière et al., 2002; Techer et al., 2014; Fareid et al., 2025).

### 2.9 Antioxidant efficacy of methanolic *A. vulgaris* extract

The antioxidant properties of methanolic *A. vulgaris* extract were assessed through two assays as follows:

#### 2.9.1 DPPH assay

The antioxidant efficacy of methanolic *A. vulgaris* extract was evaluated through the DPPH radical scavenging method, as outlined in the study conducted by Shehata et al. (2024). The extract was analyzed at various concentrations: 1,000, 500, 250, 125, 62.5, 31.25, 15.62, and 7.81 μg/mL. A 0.1 mmol/L ethanol solution of DPPH was introduced to 5 mL of the sample, which was then mixed thoroughly. As a standard control, ascorbic acid was incorporated into experimental design. The mixture was allowed to rest for 20 min at 27 °C, after which the absorbance was measured at 517 nm. The IC_50_ values for both ascorbic acid and *A. vulgaris* extract were calculated, indicating the concentration required to achieve a 50% reduction in the initial DPPH concentration. The antioxidant capacity of methanolic *A. vulgaris* extract was assessed using the specified equation. To evaluate the DPPH scavenging activity, the following formula was applied: (Absorbance of ascorbic acid control – Absorbance of *A. vulgaris* extract)/Absorbance of ascorbic acid control × 100%. This experiment was performed in triplicate, and the results were reported as the mean value ± standard deviation.

#### 2.9.2 ABTS assay

The antioxidant activity of methanolic *A. vulgaris* extract were analyzed through the ABTS [2,2′-azino-bis (3-ethylbenzothiazoline-6-sulfonic acid)] radical decolorization technique, which was slightly modified from the methodology presented by Elbestawy et al. (2023). The concentrations of methanolic *A. vulgaris* extract and ascorbic acid used in this research were 1,000, 500, 500, 500, 250, 125, 62.5, 31.25, 15.62, and 7.81 μg/mL. An ABTS radical was formed by combining a 7 mmol/L ABTS solution with 2.4 mmol/L potassium persulfate in a dark environment for 12–16 h at 25 °C. Before the reaction began, this solution was diluted in ethanol at a 1:89 (V/V) ratio and allowed to stabilize at 30 °C, with its absorbance recorded at 734 nm. The antioxidant activity of both the standard and *A. vulgaris* extract were determined as ABTS scavenging activity (%) through the application of the following equation:


$$\begin{array}{l} ABTS\;scavenging\;activity=\\ \frac{\text{control absorbance - different concentrations absorbance}}{\text{control absorbance}}\;X100 \end{array}$$

The experiment was carried out in triplicate, and the results were expressed as mean values ± standard deviation.

##### 2.9.2.1 Cytotoxicity study of methanolic *A. vulgaris* extract

The cytotoxic effects of methanolic *A. vulgaris* extract were assessed through the MTT assay as described by Shehata et al. (2024). The cell lines utilized, which included Vero cells and HFB4, were sourced from ATCC in Rockville. The existing adherent culture medium was replaced with a fresh medium containing varying concentrations of *A. vulgaris* extract, specifically ranging from 1,000 to 31.25 μg/mL, and the cells were incubated at 37 °C for a period of 24 h. Following the incubation period, the cells underwent three washes with either fresh medium or cold phosphate-buffered saline (PBS) and were subsequently treated with 0.5 mg/mL MTT solution for a time frame of 2–5 h. Upon completion of the MTT treatment, the solution was discarded, and 200 μL of dimethyl sulfoxide (DMSO) was introduced to each well to facilitate the dissolution of the formazan crystals. The optical density (OD) was then recorded at 570 nm, and the percentages of cell viability and cell death were computed using the relevant formulas:


$$\begin{array}{l} \text{Cell viability }(\%)=(\text{OD of treated cells/OD of control cells})\\ \;\times \;\text{100}\\ \text{ Cell death }(\%)=[(\text{OD of control }-\text{OD of sample})\text{/OD of}\\ \text{ control}]\times \;100. \end{array}$$

Three repetitions of the experiment were performed, and the results were reported as mean ± standard deviation.

### 2.10 Characterization of the methanolic *A. vulgaris* extract by GC-MS

The methanolic extract of *A. vulgaris* was subjected to analysis and identification through GC-MS spectroscopy, following the protocol established by El-Sherbiny et al. (2022), with minor modifications. The extract was prepared using analytical grade methanol. The GC-MS analysis was performed utilizing a Thermo Scientific trace GC1310-ISQ mass spectrometer (Austin, Texas, USA), which was equipped with a direct capillary column measuring 25 mm in length, 0.25 μm in thickness, and possessing an internal diameter of 30 mm. A 1 μl sample was injected at a temperature of 250 °C, with a helium to sample ratio of 1:30 acting as the carrier gas. The oven temperature was held at 50 °C for 5 min before being increased to 230 °C at a rate of 5 °C per minute, where it was maintained for an additional 2 min. The mass spectrometer functioned in electron ionization (EI) mode at 200 °C and 70 eV, scanning a range from 40 to 1,000 m/z. The resulting spectra were compared against those of compounds cataloged in the NIST 11 and WILEY 09 libraries (Wiley, New York, NY, USA).

### 2.11 Characterization of methanolic *A. vulgaris* extract by HPLC

#### 2.11.1 HPLC sample preparation

The methanolic *A. vulgaris* extract was evaluated using a validated HPLC method. Ten milliliters of 50% methanol were used to dissolve 100 milligrams of the extract, which had been precisely measured. After passing through a nylon membrane filter with a pore size of 0.2 μm, the solution was put through a triple analysis.

#### 2.11.2 HPLC equipment and chromatographic parameters

The extract of *A. vulgaris* was introduced into a HPLC system (Shimadzu SPD-10 A, Kyoto, Japan), which comprised a SIL-10AD auto-injector, an SPD-10AV UV-Vis detector (280 nm), a DGU-10 A degasser, and an LC-10AD pump. For the separation process, a Shim-pack CLC-ODS (C-18) (2 cm, 4.6 mm, 5 μm) sourced from Cheshire, UK was utilized, in conjunction with a C18 guard column. The elution was executed using a gradient solvent system consisting of 1% acetic acid (solvent A) and acetonitrile (solvent B) as the mobile phases. The gradient was defined as A (H2O: AA 94:6, pH = 2.27), B (ACN 100%), with the intervals of 0–15 min indicating 15% B, 15–30 min indicating 45% B, and 30–45 min indicating 100% B. The flow rate was maintained at one milliliter per minute at room temperature. The peak width for each compound's HPLC analysis was taken into account (El-Sherbiny et al., 2024).

### 2.12 Statistical analysis

The experiments were conducted in triplicate, and the mean ± standard deviation was used to present the results. To compare the experimental and control groups, a two-way ANOVA was used. To identify statistically significant differences (*p*-value < 0.05), statistical analyses were performed using GraphPad Prism Software version 8.0 (GraphPad Software, Inc., La Jolla, CA, USA).

## 3 Results

### 3.1 Prevalence of genes encoding enterotoxins and emetic toxins among *B. cereus* isolates

A total of 58 *Bacillus cereus* isolates from various sample sources were analyzed using PCR to detect the presence of five diarrheal toxin-encoding genes *hblA, hblB, nheA, nheB*, and *bceT* and one emetic toxin-encoding gene, *ces*. The results showed that the *B. cereus* isolates possessed the *nhe* and *hbl* gene complexes at frequencies of 43.09% and 29.31%, respectively. Among the enterotoxin genes, *nheA* and *nheB* were detected in 20.68% and 22.41% of the isolates, respectively, while *hblA* and *hblB* were present in 8.62% and 20.68% of the isolates, respectively. The *nhe* gene complex was the most frequently detected, with both *nheA* and *nheB* present in 31.03% (18/58) of the isolates. In comparison, both *hblA* and *hblB* were found together in only 6.89% (4/58) of the isolates. Additionally, the *bceT* and *ces* genes were identified in 29.31% and 15.51% of the isolates, respectively, as summarized in Table 2.

**Table 2:** Prevalence of enterotoxins and emetic toxin genes among *B. cereus* isolates.

**Genes**	**Pastry products (*n* = 17)**	**Cereal products (*n* = 13)**	**Spices (*n* = 12)**	**Canned products (*n* = 7)**	**Fresh-cut vegetables (*n* = 9)**	**Total (*n* = 58)**
**HBL gene**						
*hblA+*	1 (5.88)	2 (15.38)	2 (16.66)	0 (0.0)	0 (0.0)	5 (8.62)
*hblB+*	1 (11.76)	3 (23.07)	3 (24.99)	2 (28.57)	3 (33.33)	12 (20.68)
*hblA+ hblB+*	1 (5.88)	1 (7.96)	2 (16.66)	0 (0.0)	0 (0.0)	4 (6.89)
None detected	14 (88.22)	7 (53.83)	5 (41,66)	5 (71.42)	6 (66.66)	37 (68.78)
**NHE gene**						
*nheA+*	1 (5.88)	3 (23.07)	3 (24.99)	2 (28.57)	3 (33.33)	12 (20.68)
*nheB+*	2 (11.76)	2 (15.38)	5 (41,66)	2 (28.57)	2 (22.22)	13 (22.41)
*nheA+ nheB+*	4 (23.52)	5 (39.80)	2 (16.66)	3 (42.85)	4 (44.44)	18 (31.03)
None detected	10 (58.80)	3 (23.07)	2 (16.66)	0 (0.0)	0 (0.0	15 (25.86)
**Other genes**						
*bceT+*	4 (23.52)	3 (23.07)	3 (24.99)	5 (71.42)	2 (22.22)	17 (29.30)
*ces+*	2 (11.76)	5 (39.80)	2 (16.66)	0 (0.0)	0 (0.0)	9 (15.51)

### 3.2 Antimicrobial susceptibility of *B. cereus* isolates

The sensitivity of *Bacillus cereus* isolates to fifteen different antibiotics was evaluated using the disc diffusion method. The results showed that the isolates exhibited high sensitivity to rifampicin (96.54%), ciprofloxacin (91.37%), and fusidic acid (87.92%). In contrast, most isolates demonstrated resistance to ampicillin (86.20%), novobiocin (65.51%), and oxacillin (62.06%), as shown in Table 3. Multidrug resistance (MDR) was detected in 24.13% (14/58) of the *B. cereus* isolates.

**Table 3:** Antibiotics susceptibility of *B. cereus* isolates.

**Antibiotics**	**Conc. (μg/disc)**	**No. of strains (%)**		
		**Resistant**	**Intermediate**	**Susceptible**
Ampicillin	10	50 (86.20)	3 (5.17)	5 (8.20)
Oxacillin	1	36 (62.06)	10 (17.24)	12 (20.68)
Erythromycin	15	13 (22.41)	9 (15.51)	36 (62.06)
Chloramphenicol	30	3 (5.17)	6 (10.34)	49 (84.47)
Novobiocin	30	38 (65.51)	6 (10.34)	14 (24.13)
Nitrofuran	50	12 (20.68)	5 (8.20)	41 (70.68)
Ciprofloxacin	5	3 (5.17)	2 (3.44)	53 (91.37)
Streptomycin	10	5 (8.20)	3 (5.17)	50 (86.20)
Gentamycin	10	22 (37.92)	6 (10.34)	30 (51.72)
Vancomycin	30	3 (5.17)	5 (8.20)	50 (86.20)
Rifampicin	5	2 (3.44)	0.0	56 (96.54)
Kanamycin	30	4 (6.89)	5 (8.20)	47 (81.02)
Tetracycline	30	6 (10.34)	12 (20.68)	40 (68.96)
Fusidic acid	10	1(1.72)	5 (8.20)	51 (87.92)
Amikacin	30	2 (3.44)	6 (10.34)	50 (86.20)

### 3.3 Antibacterial activity of *A. vulgaris* extracts against *B. cereus* isolates

**Figure 1:**
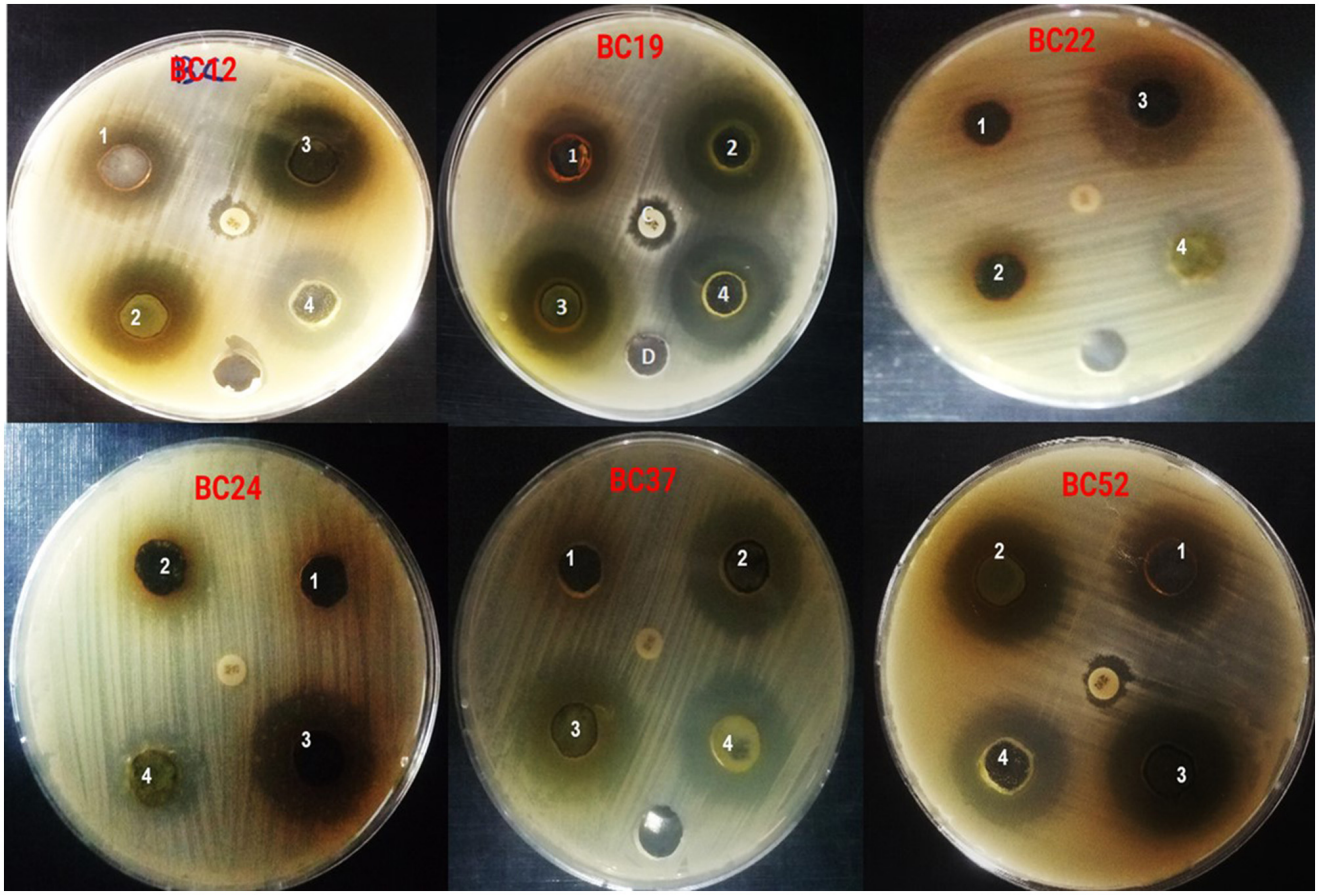
Antibacterial activity of *A. vulgaris leaves* extracts against multidrug *B. cereus* isolates (1) water extract (2) ethyl acetate extract, (3) methanol extract, (4) chloroform extract and gentamicin as a positive control.

The *A. vulgaris* extracts demonstrated considerable antibacterial activity against various *B. cereus* isolates. The results showed that the aqueous extract of *A. vulgaris* produced inhibition zones ranging from 10.05 ± 0.41 mm to 15.06 ± 0.23 mm, the ethyl acetate extract from 10.07 ± 0.13 mm to 23.12 ± 0.40 mm, the methanol extract from 19.20 ± 0.25 mm to 27.10 ± 0.13 mm, and the chloroform extract from 11.30 ± 0.20 mm to 23.00 ± 0.17 mm. In comparison, gentamicin, used as a positive control, showed no activity against the isolates. Among all extracts, the methanolic extract exhibited the highest antibacterial activity, as shown in Table 4 and Figure 1.

**Table 4:** Antibacterial action of *A. vulgaris* extracts against MDR *B. cereus*.

**Bacterial strains**	**Mean of inhibition zone diameter mm (mean** ±**SD)**				
	**Water**	**Ethyl acetate**	**Methanol**	**Chloroform**	**Gentamicin**
*B. cereus* 12	10.05± 0.41	12.10 ± 0.23	20.21 ± 0.12	13.14± 0.20	0.0
*B. cereus* 19	11± 0.57	21± 0.27	24± 0.32	23± 0.17	0.0
*B. cereus* 22	0.0	10.07± 0.13	19.20 ± 0.25	0.0	0.0
*B. cereus* 24	0.0	0.0	26.5± 0.20	11.3± 0.20	0.0
*B. cereus* 37	0.0	23.12± 0.41	22.14± 0.24	20.11± 0.40	0.0
*B. cereus* 52	15.06± 0.23	21.10 ± 0.21	27.10± 0.13	19.16± 0.18	0.0

### 3.4 MIC of methanolic of *A. vulgaris* extract against MDR *B. cereus*

**Figure 2:**
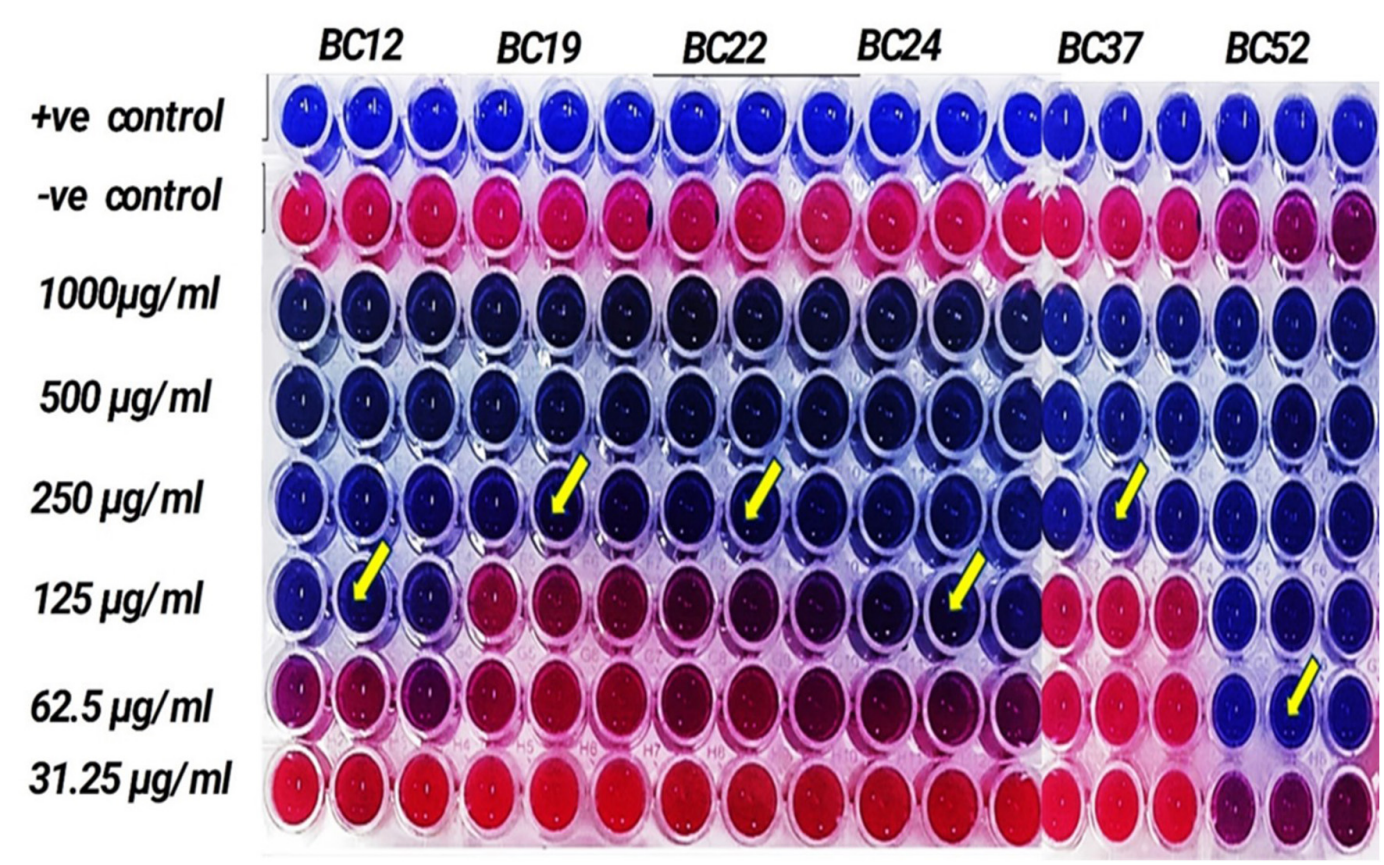
MIC of methanolic *A. vulgaris* leaves extract against MDR *B. cereus*.

Due to the methanolic extract of *A. vulgaris* exhibiting higher antibacterial activity compared to the other solvent extracts, it was selected for minimum inhibitory concentration (MIC) assessment. The findings indicated that the methanolic *A. vulgaris* extract exhibited MIC values ranging from 62.5 to 250 μg/ml against multidrug-resistant (*MDR*) *B. cereus* isolates, compared to ciprofloxacin, which showed MIC values ranging from 2.0 to 10.0 μg/ml, as detailed in Table 5 and Figure 2.

**Table 5:** MIC of methanolic *A. vulgaris* extract against MDR *B. cereus* isolates.

**Bacterial strains**	**Methanolic extract (μg/mL)**	**Ciprofloxacin (μg/mL)**
*B. cereus* 12	125	5.0
*B. cereus* 19	250	7.5
*B. cereus* 22	250	10.0
*B. cereus* 24	125	5.0
*B. cereus* 37	250	10.0
*B. cereus* 52	62.5	2.0

### 3.5. Antibiofilm activity of methanolic *A. vulgaris* extract against MDR *B. cereus*

**Figure 3:**
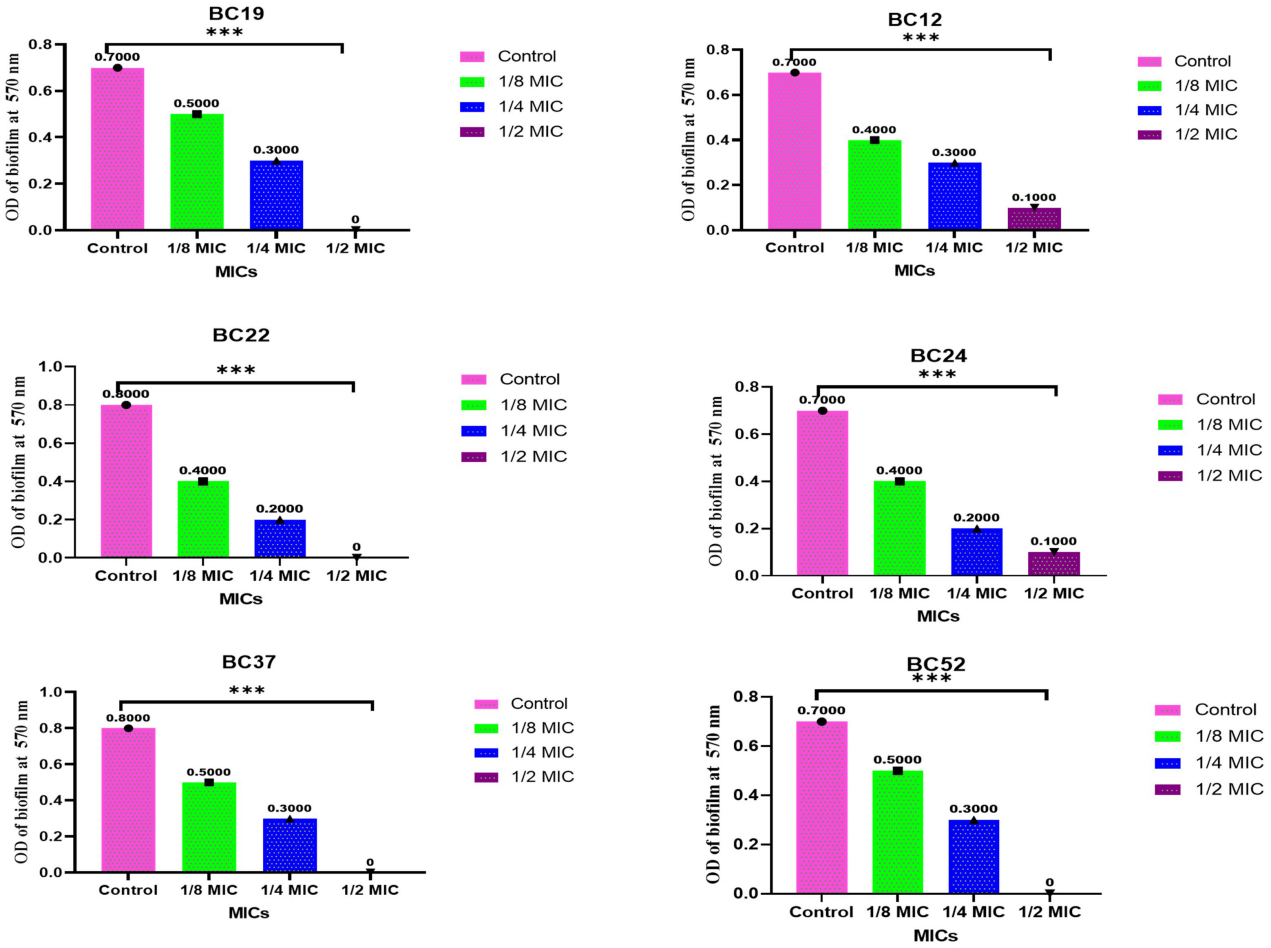
Antibiofilm activity of methanolic *A. vulgaris* extract against MDR *B. cereus* isolates (***p ≤ 0.0001).

The antibiofilm efficacy of the methanolic *A. vulgaris* leaf extract demonstrated significant anti-biofilm activity against MDR *B. cereus* isolates at concentrations of 1/8, 1/4, and 1/2 MIC. Notably, biofilm formation by isolates 19, 24, 37, and 52 was completely inhibited at the 1/2 MIC concentration, as shown in Figure 3.

### 3.6 Effects of methanolic *A. vulgaris* extract on expression toxigenic and emetic genes in *B. cereus* isolates

**Figure 4:**
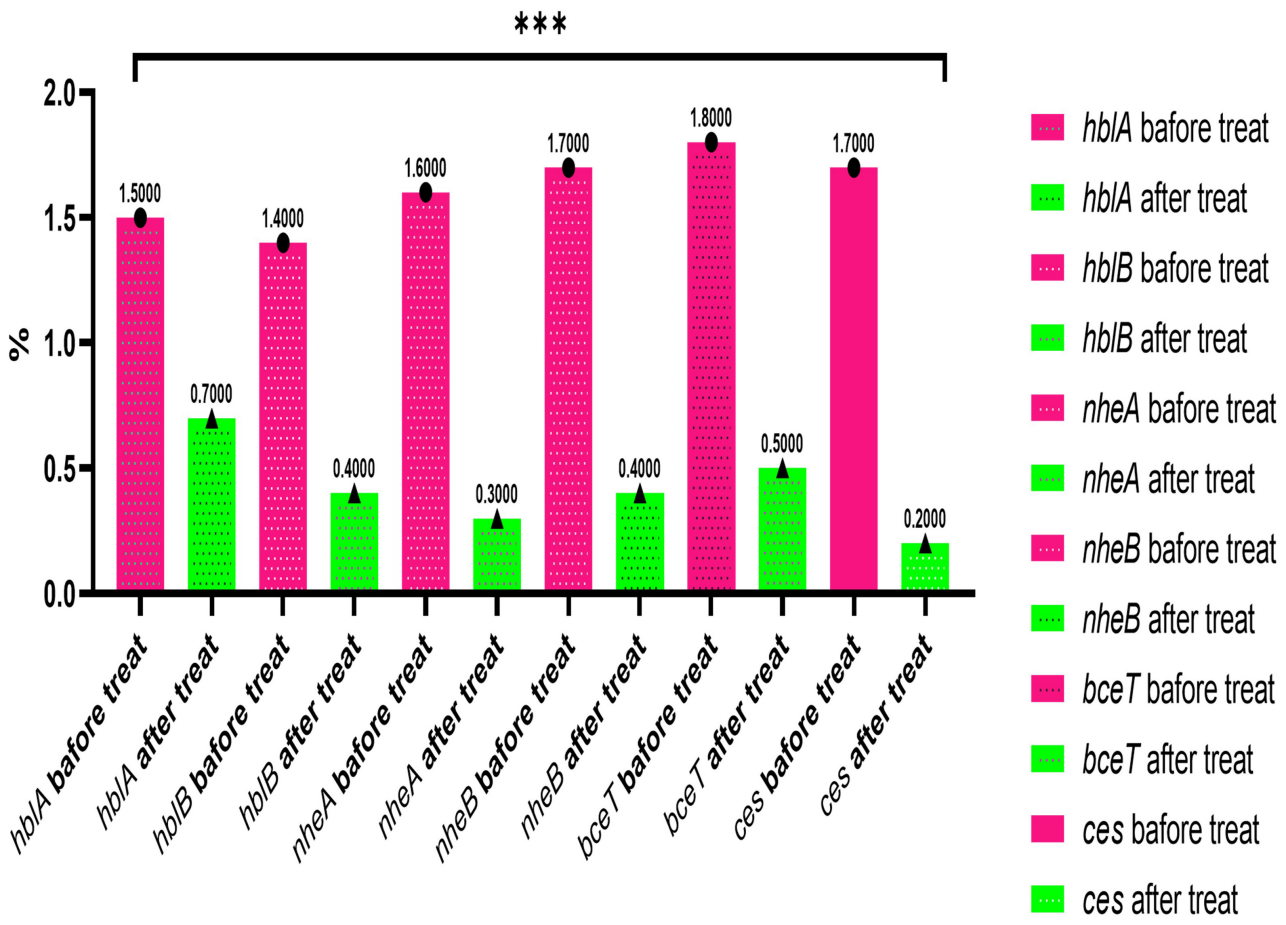
qRT-PCR expression of toxigenic and emetic genes (*hblA, hblB, nheA, nheB, bce*T, and *ces*) in *B. cereus* treated with ½ *A. vulgaris* leaves methanolic extract and untreated (***p ≤ 0.0001).

The findings reveal that *B. cereus* strains treated with the methanolic *A. vulgaris* extract at half the MIC for an overnight period showed a significant reduction in the expression levels of the *hblA, hblB, nheA, nheB, bceT*, and *ces* genes, with a fold change ranging from −2.5 to −5.2 (^***^P < 0.0001) compared to the untreated control isolates. This result suggests that the methanolic *A. vulgaris* extract contributes to the downregulation of toxigenic genes, as illustrated in Figure 4.

### 3.7 Antioxidant activity methanolic *A. vulgaris* leaves extract

**Figure 5:**
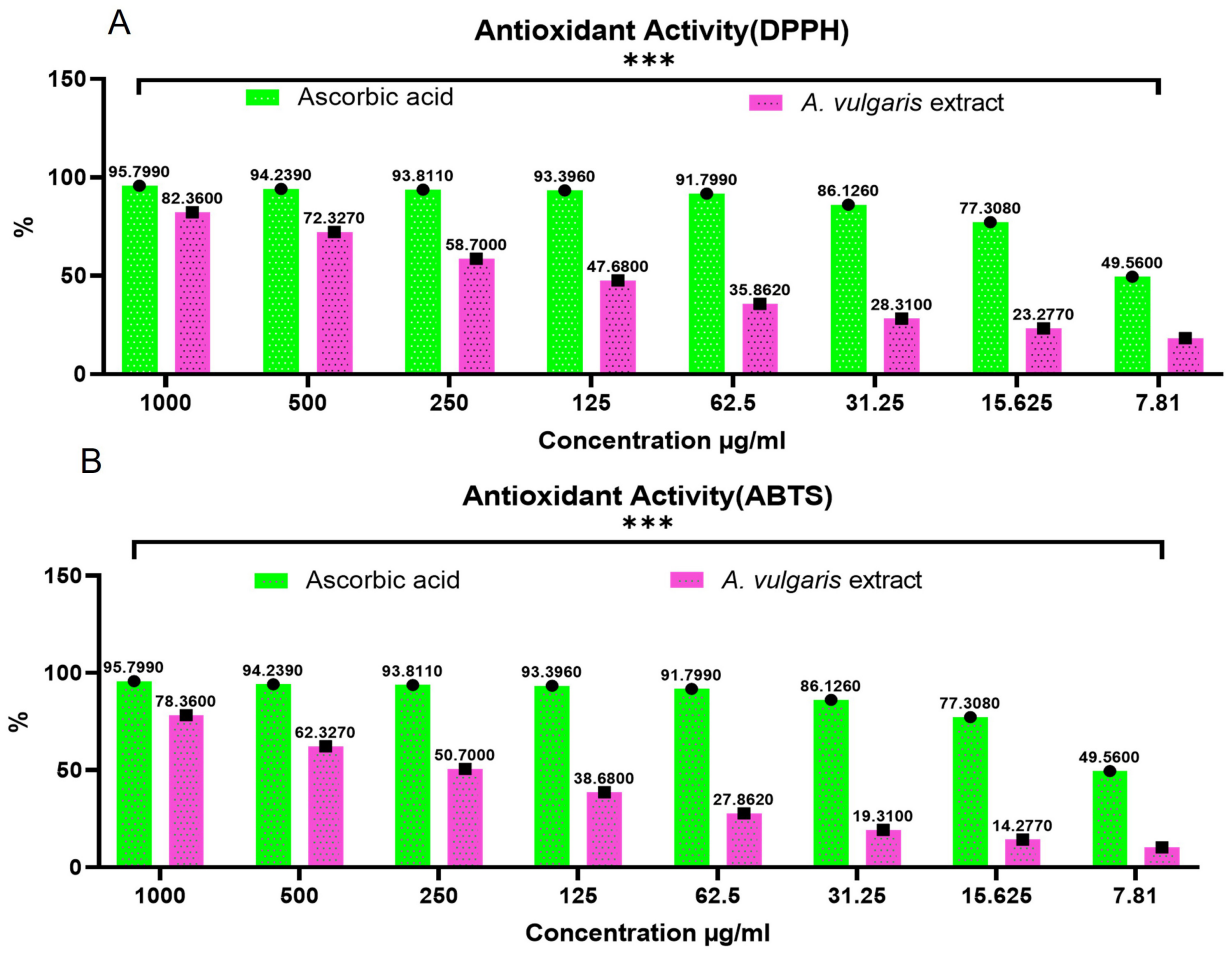
Antioxidant activity of *A. vulgaris* leaves extract, **(A)** DPPH and **(B)** ABTS (***P <0.0001).

In this study, DPPH and ABTS assays were employed to evaluate the antioxidant potential of the methanolic *A. vulgaris* extract and its ability to scavenge free radicals. The DPPH assay revealed that the methanolic *A. vulgaris* extract exhibited an antioxidant capacity with an IC50 value of 12.7 μg/mL, compared to 8.9 μg/mL for ascorbic acid. Similarly, the ABTS assay yielded IC50 values of 14.2 μg/mL for the extract and 7.61 μg/mL for ascorbic acid, as shown in Figures 5A, B. These findings indicate that while the *A. vulgaris* extract possesses antioxidant activity, it is significantly less effective than ascorbic acid in scavenging free radicals.

### 3.8 Cytotoxic activity of methanolic *A. vulgaris* extract against Vero and HFB4 cells

**Figure 6:**
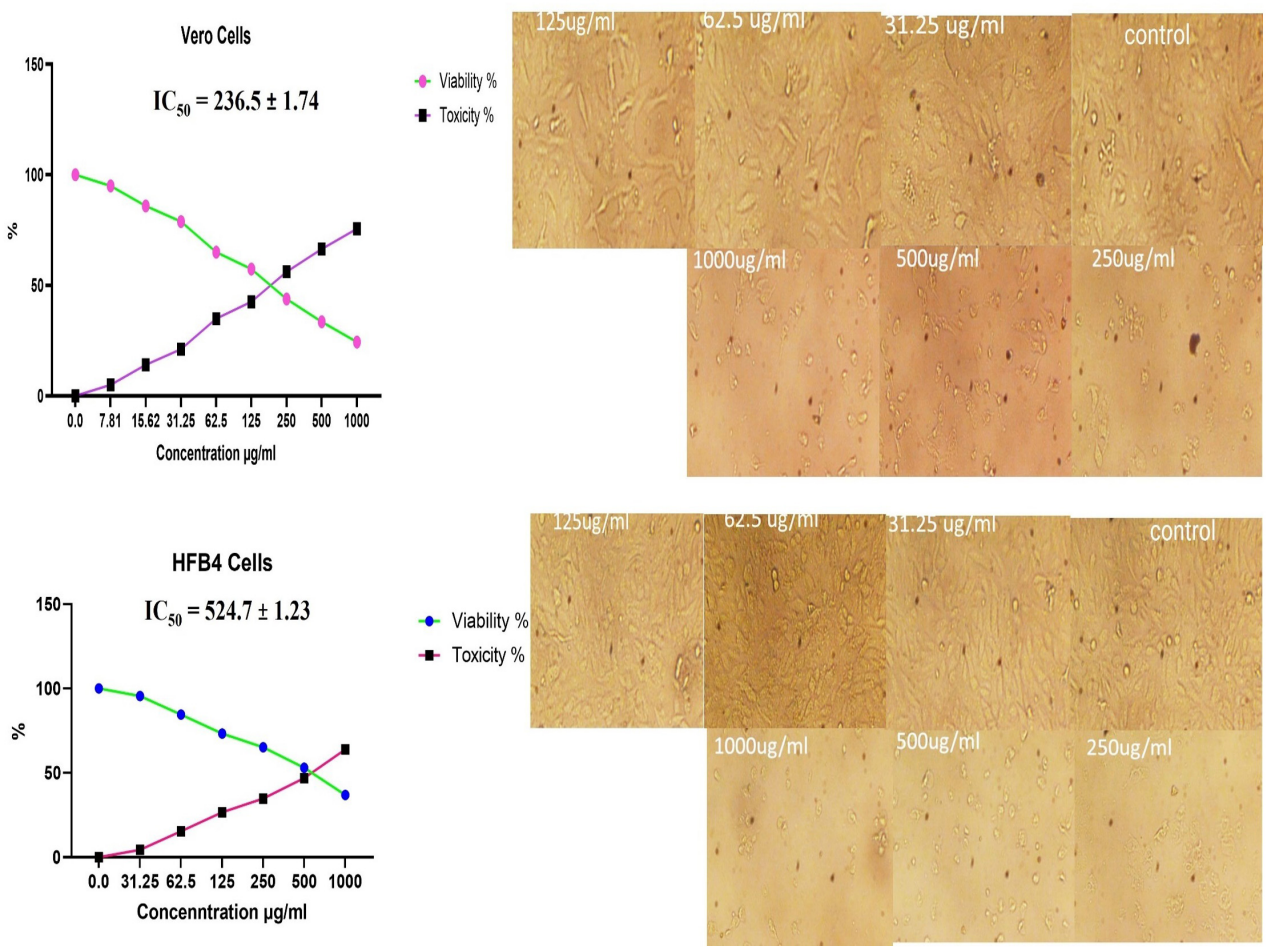
Cytotoxicity effects of methanolic *A. vulgaris* leaves extract on Vero and HFB4 cells lines.

The treatment of Vero and HFB4 cell lines with the methanolic *A. vulgaris* extract at various concentrations for 24 h resulted in minor morphological changes on the cell surface and within the cytoskeleton, which were observed in relation to cell viability compared to the control group. The MTT assay results also showed a significant reduction in cell viability starting at concentrations of 200 μg/mL and above. The IC50 values indicating the concentration required to inhibit 50% of cell viability were determined to be 236.5 ± 1.74 μg/mL for Vero cells and 524.7 ± 1.23 μg/mL for HFB4 cells, as illustrated in Figure 6. These results indicate that the methanolic *A. vulgaris* extract is safe at low concentrations.

### 3.9 Identification of methanolic *A. vulgaris* extract components by HPLC and GC-MS analysis

**Figure 7:**
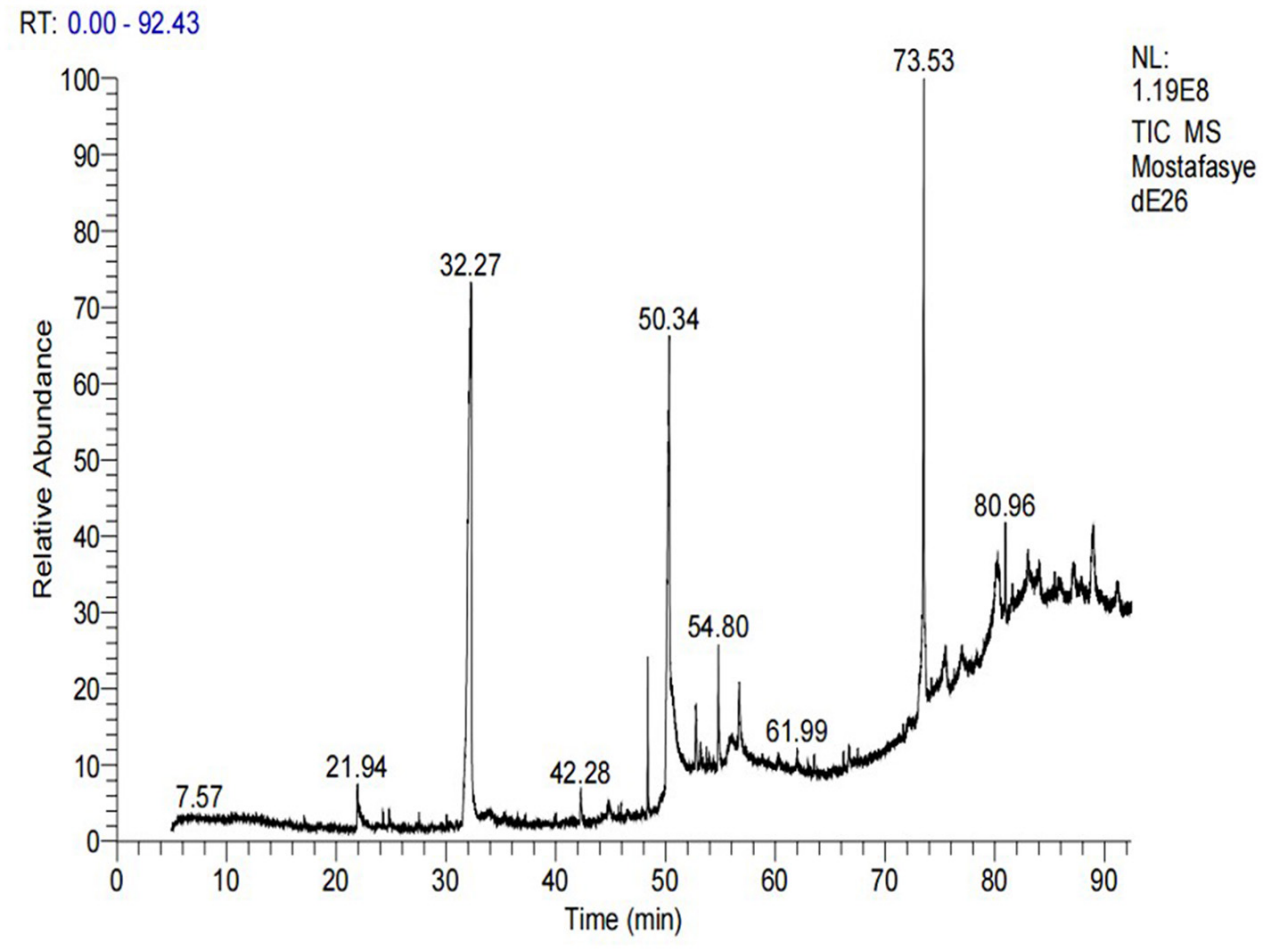
GC-MS chromatogram of methanolic *A. vulgaris* extract.

GC-MS analysis of the methanolic *A. vulgaris* extract identified fifteen compounds, with dopamine N, N-dimethyl-dimethyl ether (40.31%) being the most abundant, followed by *n*-hexadecanoic acid (16.57%) and 5,11,17,23-tetrakis (15.30%), as shown in Figure 7 and Table 6. HPLC analysis using a UV-Vis diode array detector (DAD) was employed to identify and quantify phenolic compounds and flavonoids present in the methanolic extract of *A. vulgaris* leaves. This analysis was based on peak area percentage and retention time (RT), as presented in Table 7. Among the detected polyphenolic compounds, the major constituents were chlorogenic acid (39.13% at RT 4.51 min), gallic acid (11.62% at 3.14 min), caffeic acid (1.23% at 5.07 min), coumaric acid (8.40% at 8.91 min), ferulic acid (1.22% at 9.50 min), and cinnamic acid (0.18% at 13.14 min).For flavonoids, quercetin showed a prominent peak at RT 2.72 min with a relative abundance of 15.13%, followed by luteolin (17.45% at 3.94 min), pinocembrin (7.24% at 0.85 min), rhamnetin (4.34% at 6.75 min), chrysin (2.31% at 10.21 min), and hesperidin (0.31% at 14.59 min).

**Table 6:** Chemical profiling of methanolic *A. vulgaris e*xtract by GC-MS spectrometry.

**Peak**	**Rotation time**	**Contents %**	**Compound**	**Molecular formula**	**Molecular weight**
1	21.94	1.25	2-Cyclohexen-1-one,3-methyl-6-(1-methylethyl)-	C _10_H_10_ O	152.23
2	32.28	40.31	Dopamine, N, N-dimethyl-dimethyl ether	C_12_H_19_NO_2_	209.28
3	48.39	3.05	Hexadecanoic acid, methylester	C_17_H_34_O_2_	270.45
4	50.33	16.57	n-Hexadecanoic acid	C_16_H_32_O_2_	256.42
5	52.76	2.03	Pallensin	C_15_H_20_O_4_	264.31
6	54.80	3.00	Methyl stearate	C_19_H_38_O_2_	298.50
7	56.70	2.30	Octadecanoic acid	C_18_H_36_O_2_	284.47
8	73.52	15.30	5,11,17,23-Tetrakis	C_44_H_54_O_4_	646.9
9	73.57	1.67	18,19-Secoyohimban-19-oic acid,	C_21_H_28_N_2_O_2_	340.5
10	75.51	2.62	5,11,17,23-Tetrakis	C_44_H_54_O_4_	646.9
11	80.10	1.92	Propanoic acid,	C_3_H_6_O_2_	74.08
12	80.16	0.84	Propanoic acid, 2-(3-acetoxy-4,4,14-trimethyla ndrost-8-en-	C_27_H_42_O_4_	430.6
13	80.23	3.30	4H-1-Benzopyran-4-one	C_9_H_6_O_2_	146.14
14	80.96	2.33	Cholestane-3,5-diol, 5-acetate, (3á,5à)-	C_29_H_50_O_3_	446.7
15	88.94	3.52	1H-Purin-6-amine, (N-(3-fluorophenyl) methyl)-	C_12_H_10_FN_5_	243.24

**Table 7:** HPLC analysis of methanolic extract *A. vulgaris* leaves and its biological activities.

**No**.	**RT**	**%**	**Name/molecular formula**	**Chemical structure**	**Biological activities**	**References**
1	2.721	15.138	Quercetin C15H10O7	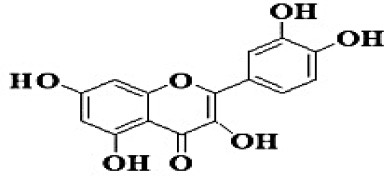	Antibacterial, antioxidant, antiviral, anti-inflammatory, anticancer, antihypertensive, and vasodilator activity.	El-Sherbiny et al., 2024
2	3.145	11.624	Gallic acid C7H6O5	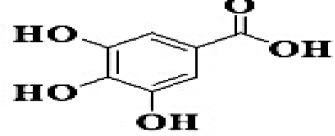	Antibacterial, antioxidant, anti-inflammatory, anticancer antidiabetic, anti-obesity,	El-Sherbiny et al., 2024
3	3.940	17.454	Luteolin C1_5_H1_0_O_6_	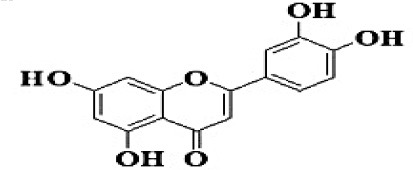	Antibacterial, antioxidant, anti-inflammatory, anticancer and liver and nervous system protection	Zhu et al., 2024
4	4.510	39.138	Chlorogenic acid C16H18O9	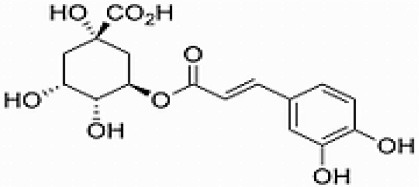	Antibacterial, antioxidant, anti-inflammatory, anticancer and liver, kidney and nervous system protection	El-Sherbiny et al., 2024
5	5.078	1.232	Coffeic acid C9H8O4	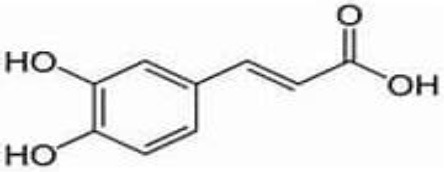	Antioxidant, anti-inflammatory, anticancer, antibacterial, antiviral, and Parkinson's Disease	El-Sherbiny et al., 2024
6	6.759	4.342	Rhamnetin C_16_H_12_O_7_	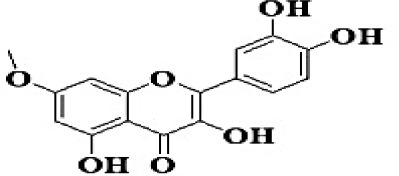	Anti-inflammatory, cardio-protective, antioxidant, neuroprotective, antibacterial, antiviral, and antiparasitic.	Medeiros et al., 2022
7	7.243	0.856	Pinocembrin C_15_H_12_O_4_	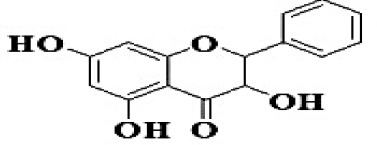	Antidiabetic, antibacterial, antiparasitic, antiviral, antioxidant and anti-inflammatory	Rasul et al., 2013
8	8.916	8.476	Coumaric acid C_9_H_8_O_3_	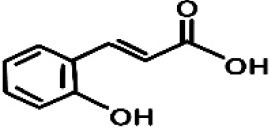	antioxidants, anti-inflammatory, anti-aging, immunomodulatory, antidiabetic, antibacterial, antiparasitic, and antiviral	El-Sherbiny et al., 2024
9	9.50	1.223	Ferulic acid C_10_H_10_O_4_	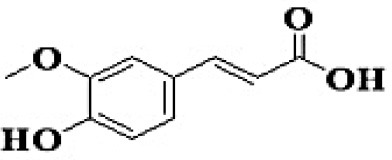	Antioxidant, anti-inflammatory, cardio-protective, immunomodulatory, antidiabetic, antiviral, antibacterial, and antiparasitic	El-Sherbiny et al., 2024
10	10.21	2.315	Chrysin C_15_H_10_O_4_	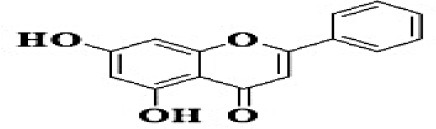	Antioxidant, anti-inflammatory and anticancer, antibacterial and antiviral	Mani and Natesan, 2018
11	13.14	0.188	Cinnamic acid C_9_H_8_O_2_	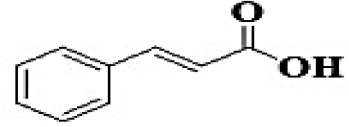	Antioxidant, anti-inflammatory, anticancer, antibacterial and antiviral	El-Sherbiny et al., 2024
12	14.59	0.311	Hesperidin C_28_H_34_O_15_	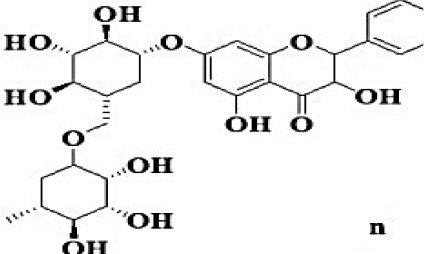	Anti-inflammatory, antioxidant, antitumor and antibacterial	Ji et al., 2024

## 4 Discussion

This study emphasized the toxigenic potential of *B. cereus* strains isolated from various food samples. The results underscore the importance of monitoring and controlling these pathogens within the food supply to protect public health and prevent foodborne illnesses. The findings demonstrated that *B. cereus* isolates exhibit multi-toxigenicity, harboring at least one gene from both the NHE and HBL complexes, in addition to the *bceT* and *ces* genes. These observations are consistent with previous findings reported by Gdoura-Ben Amor et al. (2019). Furthermore, Tewari et al. (2015) found that 55.2% and 89.7% of *B. cereus* strains tested positive for at least one of the HBL and NHE genetic determinants, respectively. Our analysis also showed that genes associated with the HBL complex were present at a lower frequency than those of the NHE complex, consistent with earlier studies by Tewari et al. (2015) and Castiaux et al. (2016), which reported that HBL-related genes are less prevalent than NHE-related ones. The occurrence of *hblA* and *hblB* genes aligns with findings by Ceuppens et al. (2011), who observed that 84–100% of *B. cereus* group strains carried *nhe* genes, while *hbl* genes were detected in 29–92% of the isolates they examined. In our study, both genes of the NHE complex were detected in 31.03% (18/58) of the isolates, while 6.89% (4/58) carried the complete HBL gene complex. These results disagree with those reported by Ngamwongsatit et al. (2008), who found that none of the 411 *B. cereus* strains evaluated harbored one or more genes from the HBL or NHE complexes. This may be attributed to the inability to detect all genes by PCR in most isolates, as well as the presence of polymorphisms in the sequences of the HBL and NHE complex genes, rather than their complete absence (Guinebretiere et al., 2006). In this study, the *bceT* and *ces* genes were detected in 29.30% and 15.51% of *B. cereus* isolates, respectively. The occurrence of emetic intoxication genes is often associated with starchy foods such as rice, noodles, pasta, and mashed potatoes (Agata et al., 2002; Naranjo et al., 2011). In addition, consistent with our findings, López et al. (2015) and Messelhäusser et al. (2014) reported the identification of emetic *B. cereus* group strains in cooked chicken, as well as in foods such as soups, sauces, and mixed or buffet meals. However, none of the *B. cereus* group strains isolated from spices contained the emetic toxin gene (*ces*) in studies conducted by Hariram and Labbé (2015) and Fogele et al. (2018).

Foodborne pathogens pose a significant global challenge, serving as reservoirs of infection and contributing to the development of antimicrobial resistance. Our investigation revealed that *B. cereus* isolates exhibited varying levels of susceptibility to the antimicrobial agents tested. The majority of *B. cereus* isolates were resistant to ampicillin, consistent with previous studies reporting notable resistance of this group to β-lactam antibiotics (Gdoura-Ben Amor et al., 2019; Lee et al., 2012; Park et al., 2009). This may be attributed to the strains' ability to produce β-lactamases-enzymes that play a crucial role in the degradation of antibiotics (Park et al., 2009). In the context of the *B. cereus* group, β-lactamase production can confer resistance even to third-generation cephalosporins (Özcelik and Citak, 2009). In our study, 65.5% of the strains were resistant to novobiocin. In contrast, Aklilu et al. (2016) reported that all *B. cereus* group isolates they tested were resistant to novobiocin. In our study, more than 96%, 91%, and 87% of *B. cereus* isolates were sensitive to rifampicin, ciprofloxacin, and fusidic acid, respectively. These findings are consistent with results reported by Gdoura-Ben Amor et al. (2019) who demonstrated that all *B. cereus* isolates were fully sensitive to rifampicin, chloramphenicol, ciprofloxacin, and gentamicin. Furthermore, Banerjee et al. (2011) reported that 100% of *B. cereus* isolates were sensitive to ciprofloxacin in patient samples, and other studies have produced similar results when evaluating sensitivity to ciprofloxacin in food samples (Aklilu et al., 2016; Oladipo and Adejumobi, 2010; Jensen et al., 2001). In the current study, MDR was identified in 24.13% (14/58) of the *B. cereus* isolates. In the previous study, an analysis of the susceptibility *B. cereus* isolates revealed that only two strains were non-resistant to 15 antibiotics, while the majority strains demonstrated resistance to three or more antibiotics. Additionally, certain reports have indicated the presence of carbapenem-resistant *B. cereus* bacteremia, and some *in vitro* investigations have revealed that *B. cereus* genetically harbors metallo-beta lactamase activity, which contributes to its antibiotic resistance profile. This alarming trend underscores the importance of continuous monitoring and the development of new therapeutic strategies to combat infections caused by this pathogen (Ikeda et al., 2015; Cha et al., 2023).

Natural substances obtained from plants have long been used in the pharmaceutical, food, and cosmetics industries, with documentation found in multiple nations. Ancient cultures relied on these products for both nutritional and therapeutic applications. In the past few decades, there has been a marked surge in researchers' interest in analyzing the specifics of their compositions and investigating their potential applications in diverse fields (El-Sherbiny and Elbestawy, 2022). We utilize the natural compounds available to us to tackle the increasing prevalence of diseases like cancer, diabetes, obesity, heart attacks, microbial infections, accelerated skin aging, and other new health challenges (El-Sherbiny et al., 2024). The application of natural materials offers notable benefits over medications that are derived from synthetic sources. Because of their limited side effects, this strategy is often viewed as the most advantageous when assessing the toxicological and pharmacological properties of these compounds in comparison to those from chemical origins. A broad spectrum of pharmacological effects has been discovered in extracts of natural products, which include antibacterial, antioxidant, antidiabetic, anti-inflammatory, cardio-protective, neuroprotective, immunomodulatory, antiparasitic, and antiviral functions. Different plant parts, such as seeds, roots, stems, bark, leaves, flowers, and fruits, each have unique phytochemical compositions and potential medicinal advantages (Elbestawy et al., 2023; El-Sherbiny et al., 2022). In this study, extracts derived from *A. vulgaris* leaves exhibited notable antibacterial activity against MDR *B. cereus* isolates, with the methanolic extract being the most active, showing MIC values ranging from 62.5 to 250 μg/mL. Recent investigations have revealed that *A. vulgaris*, a key therapeutic plant species in the *Artemisia* genus, possesses a variety of properties, including antioxidant, hypolipidemic, antispasmodic, analgesic, estrogenic, cytotoxic, antibacterial, antifungal, hypotensive, and broncho-lytic activities (Singh et al., 2023; Bordean et al., 2023). This suggests the potential of *A. vulgaris* as a natural antimicrobial agent for combating foodborne *B. cereus*. Furthermore, several investigations have documented the antibacterial effects of *A. vulgaris* extract on a wide range of bacterial species, including *Acinetobacter calcoaceticus, B. cereus, B. subtilis, Enterobacter faecalis, Escherichia coli, Klebsiella pneumoniae, Proteus vulgaris, Salmonella typhimurium, Serratia marcescens, Staphylococcus aureus*, and *Escherichia coli*. This implies that *A. vulgaris* extract could be an important resource in the formulation of new antibacterial treatments, especially given the rising challenge of antibiotic resistance (Oyedemi and Coopoosamy, 2015; Gou et al., 2023).

Our findings showed that the methanolic extract from *A. vulgaris* exhibited a partial inhibitory effect on biofilm formation by *B. cereus* isolates at concentrations of 1/8 and ¼ MIC, while at ½ MIC, it completely inhibited biofilm formation in some *B. cereus* isolates. These results are consistent with previous studies reporting the effectiveness of plant extracts in suppressing biofilm formation among various bacterial species (Shehata et al., 2024; El-Sherbiny et al., 2022). Furthermore, essential oils from *Artemisia dracunculus* have been shown to effectively inhibit biofilm formation in *S. Typhimurium* (*P* < 0.001) at concentrations of ½ and ¼ MIC (Mohammadi Pelarti et al., 2021). In the current study, PCR was used to investigate the effect of methanolic *A. vulgaris* extract on the expression of toxigenic and emetic genes in *B. cereus* isolates. The results indicate that *A. vulgaris* extract downregulated several diarrheal toxin-encoding genes (*hblA, hblB, nheA, nheB, bceT*, and *ces*) by −2.5- to −5.2-fold (*P* < 0.0001) at ½ MIC. Similarly, a previous study by Mohammadi Pelarti et al. (2021). demonstrated that essential oils extracted from *Artemisia dracunculus* significantly downregulated the expression of quorum sensing–related genes *luxS* and *pfs* by −4.18- and −3.12-fold, respectively, in *S. Typhimurium*, and the *hld* gene in *S. aureus* by −5.35-fold at ½ MIC.

Our investigation revealed that the methanolic extract of *A. vulgaris* exhibits significant antioxidant activity, as confirmed by both DPPH and ABTS assays. Antioxidants are known to eliminate reactive oxygen species (ROS), reduce oxidative stress, protect cells and tissues from damage, lower the risk of gout, and improve overall patient health (El-Sherbiny et al., 2024). Trinh et al. (2024) reported that the ethyl acetate fraction of *Artemisia* extract contained the highest concentrations of total polyphenols and flavonoids. This fraction also demonstrated strong antioxidant activity across several assays, including DPPH, ABTS, reducing power, and hydrogen peroxide (H_2_O_2_) scavenging. Additionally, the antioxidant properties of *A. vulgaris* were investigated by Sharmila and Aadma (2014), who used a methanolic extract in chick embryos exposed to oxidative stress. Their research showed that the presence of *A. vulgaris* enhanced the antioxidant activity of primary cells under such conditions. Another study by Haniya and Padma (2014) found that the methanolic extract exhibited the most effective free radical scavenging activity against agents such as DPPH, ABTS, hydrogen peroxide, superoxide, hydroxyl radicals, and nitric oxide. These findings further validate the strong antioxidant potential of *A. vulgaris* leaf extract. Our investigation demonstrated that the methanolic extract of *A. vulgaris* exhibits minimal cytotoxic effects on Vero and HFB4 cell lines, with IC50 values of 236.5 ± 1.74 μg/mL for Vero cells and 524.7 ± 1.23 μg/mL for HFB4 cells. These findings align with the results reported by Jakovljević et al. (2023), who observed minimal cytotoxicity of *A. vulgaris* acetone extract in SW-480 cells, in contrast to a significant cytotoxic effect from *A. alba* extract (IC50 values of 240.12 ± 25.49 μg/mL for *A. vulgaris* vs. 3.89 ± 1.47 μg/mL for *A. alba*). According to current literature, the concentration of chemical constituents in *Artemisia* extracts can vary depending on harvest time, geographic location, soil conditions, and genetic factors (Mohammadi Pelarti et al., 2021). The predominant compound identified in the methanolic leaf extract of *A. vulgaris* was dopamine N, N-dimethyl-dimethyl ether (40.31%), followed by n-hexadecanoic acid (16.57%). Earlier studies by Hammad et al. (2024) also identified dopamine N,N-dimethyl-dimethyl ether as a major plant-derived compound. Antibacterial assessments revealed significant activity against *Staphylococcus aureus* and *Pseudomonas aeruginosa*, even at low concentrations. Medicinal plants, including *Artemisia* species, have long been used to treat high fevers caused by malaria in countries such as Ghana, Mali, Nigeria, and Zambia. It has been established that *Artemisia* species contain essential oils capable of inhibiting the growth of various pathogenic bacteria, including *Staphylococcus aureus* and *Staphylococcus epidermidis* (Mohammed et al., 2022). In this study, HPLC analysis of *A. vulgaris* indicated a high content of chlorogenic acid, luteolin, and quercitrin. These results are consistent with previous literature (Melguizo-Melguizo et al., 2014; Neagu et al., 2023). The phenolic and flavonoid compounds in *A. vulgaris* extracts possess notable antidiabetic, anti-inflammatory, antioxidant, and antibacterial properties (Medeiros et al., 2022; Rasul et al., 2013; Mani and Natesan, 2018; Ji et al., 2024; Tewari et al., 2015).

## 5 Conclusion

This study highlights the toxigenic potential of *B. cereus* group strains isolated from various food samples. The findings indicate that these isolates possess significant toxigenic capabilities and antibiotic resistance, which may pose a serious public health concern. To reduce the risk of foodborne illness, it is essential to implement strict food safety protocols and increase public awareness regarding proper food handling practices. Additionally, further research is needed to explore effective strategies for controlling and preventing the spread of these resistant strains in food products. The results also demonstrated the ability of methanolic *A. vulgaris* extract to inhibit *B. cereus* isolates, suppress biofilm formation, and downregulate virulence gene expression. This suggests that *A. vulgaris* extract represents a promising alternative approach for combating *B. cereus*. Future studies may investigate the synergistic effects of *A. vulgaris* extract in various food matrices to optimize its protective potential.

## Data Availability

All data generated or analyzed during this study are included in the article.
